# Treatment of eccrine porocarcinoma with metastasis to the parotid gland using intensity-modulated radiation therapy: a case report

**DOI:** 10.1186/1752-1947-4-147

**Published:** 2010-05-22

**Authors:** Youssef H Zeidan, A Jason Zauls, Masha Bilic, Eric J Lentsch, Anand K Sharma

**Affiliations:** 1 Department of Radiation Oncology, Medical University of South Carolina, Charleston, SC, USA; 2 Department of Pathology, Medical University of South Carolina, Charleston, SC, USA; 3 Department of Otolaryngology, Medical University of South Carolina, Charleston, SC, USA

## Abstract

**Introduction:**

Cutaneous eccrine porocarcinomas are uncommon malignant tumors of the sweat gland.

**Case Presentation:**

A 76-year-old Caucasian man presented to our hospital with a left temporal mass. We describe a case of eccrine porocarcinoma with metastasis to the parotid gland with special emphasis on the role of surgical resection and adjuvant radiation therapy.

**Conclusion:**

Besides surgical resection, little is known about the role of adjuvant therapy in managing eccrine porocarcinomas. Radiation therapy should be considered within a multidisciplinary approach in patients with primary or recurrent eccrine porocarcinomas.

## Introduction

Eccrine porocarcinoma (EPC) is a rare malignant tumor of the sweat gland that accounts for 0.005% of all skin cancers [1]. Due to its low incidence, current knowledge about EPC is limited to case reports, with only 250 cases having been reported worldwide. Common EPC lesions occur on the lower extremities (50%) followed by the trunk (24%) or the head and neck (24%), with more than 50% of cases occurring in men [2]. EPC has also been reported to involve the vulva [3], penis [4] and upper extremities [5]. Although the age at presentation ranges from 19 to 90 years [6], EPC tends to affect the elderly, at an average age of 68. Surgical resection remains the gold standard for treatment. There is a 17% incidence of local recurrence and an 11% incidence of distant metastasis [7], which indicates that there is a role for adjuvant therapy.

We report the case of a patient with cutaneous EPC with secondary metastasis to the parotid gland who was treated with intensity-modulated radiation therapy after surgical resection.

## Case Presentation

**Figure 1:**
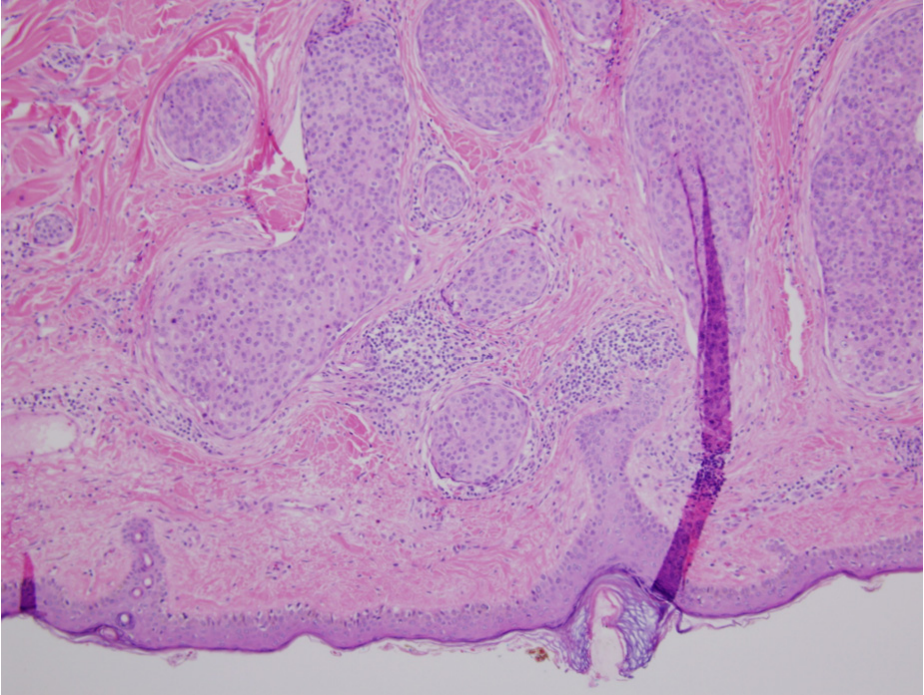
**Photomicrograph of the initial left temporal mass showing islands of polygonal tumor cells invading the dermis (hematoxylin and eosin, 10×)**.

**Figure 2:**
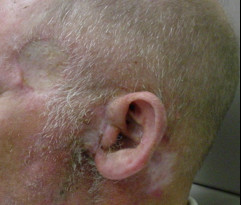
**Macroscopic view showing a new left parotid lesion and a scar at the previous frontotemporal lesion**.

**Figure 3:**
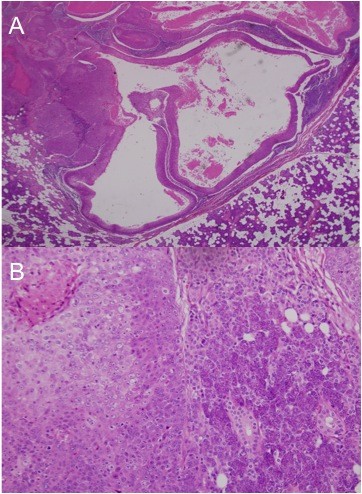
**Photomicrographs of surgical pathology specimens**. (A-B) Photomicrograph of left parotid mass showing tumor cells of similar cytology to the original temporal mass infiltrating the parotid gland. Ductal structures characteristic of eccrine porocarcinoma are seen (hematoxylin and eosin, 4× to 20×).

A 76-year-old Caucasian man presented with a long history of a left temporal lesion that had progressively enlarged only recently. Initial surgical excision yielded a mass measuring 2.5 × 2.2 × 1 cm. Histological examination revealed tumor islands infiltrating the dermis and connecting to the epidermis with a lobulated morphology (Figure 1). Since the deep resection margin was positive for malignant cells, a re-excision was performed and negative margins were verified microscopically. Eight months later, our patient presented with a 2 cm firm mass overlying the left parotid gland with minimal mobility on physical examination (Figure 2). Whole body imaging in the form of a positron emission tomography (PET) scan showed increased uptake in the left parotid gland, which was consistent with metastasis. Our patient underwent left parotidectomy with facial nerve preservation and cervical lymphadenectomy. Surgical pathology specimens revealed a moderately differentiated carcinoma with growth pattern and morphological features consistent with porocarcinoma. Microscopically, nests of polygonal malignant cells were seen infiltrating the papillary and reticular dermis (Figure 3). A surrounding dense fibrous stroma could be visualized (Figure 3). The cervical lymph nodes (14 in total) were negative.

Given the aggressive behavior of EPC described in the literature and evidence for the effective use of radiation therapy [8], our patient was offered intensity-modulated radiation therapy (IMRT). After initial consultation at the Head and Neck Radiation Oncology Clinic of the Medical University of South Carolina (MUSC) Hollings Cancer Center, a consent form was obtained. Treatment planning consisted of a contrasted head and computed tomography (CT) scan of the neck with our patient lying supine, using a head and neck thermoplast mask with bite block immobilization. Thin (3 mm) axial images were imported into the ADAC Pinnacle planning system (ADAC Laboratories, Milpitas, CA, USA). In this case, IMRT was designed using an inverse-planning algorithm.

**Figure 4:**
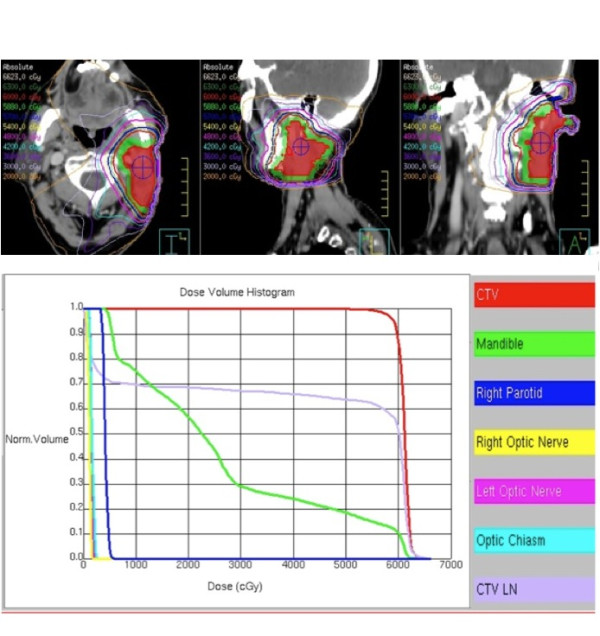
**(A) Axial, saggital and coronal CT images showing the final intensity-modulated radiation therapy (IMRT) plan and isodose distributions around the tumor bed**. Gross tumor volume (GTV) is outlined from contrast enhanced planning CT scan. Isodose lines of decreasing energies radiate out from the center of the tumor bed. The tumor is completely contained by the 95% isodose blue line (57 Gy). (B) Cumulative dose-volume histogram (DVH) of the IMRT plan. The curves illustrate the dose distribution for the clinical target volume (CTV: defined as GTV +0.5 cm), mandible, left parotid, optic nerves, optic chiasm and a cervical lymph node.

The six-beam heterogeneous plan entailed delivering a total of 60 Gy in 30 treatment fractions over six weeks. A CT scan with isodose distributions and a dose-volume histogram for the final treatment plan are shown in Figure 4. Particular attention was directed to regions of interest such as the left inner ear and optic nerve, to minimize radiation exposure well below the reported tolerance doses of 40 Gy and 50 Gy, respectively [9].

Weekly on-site treatment visits were documented during the treatment phase of our patient, followed by scheduled three-month follow-up visits. Our patient tolerated the radiation treatment well, and developed only mild xerostomia and mild paresthesias. Further follow-up revealed that our patient remains disease-free 10 months after completion of the treatment course.

## Discussion

Eccrine porocarcinoma is challenging to diagnose based on clinical presentation alone, and histopathological examination is almost always required. Typically, a patient presents with an erythematous papule with a recent change in size, bleeding or itching. The differential diagnosis includes squamous cell carcinoma (SCC), basal cell carcinoma, Paget's disease and metastatic cancer. Positive staining for periodic acid-Schiff (PAS), carcinoembryonic antigen (CEA) or angiotensin type 1 receptor can aid in making the diagnosis [10,11]. Although the etiology remains unknown, it has been suggested that EPC arises from the malignant transformation of eccrine poroma. Interestingly, an association has been proposed between EPC and the immuncompromised states such as human immunodeficiency virus (HIV), diabetes and organ transplantation [12].

Wide local excision [7] and Mohs surgery [13] are widely accepted treatment modalities for primary EPC. Surgical excision has a cure rate of 70-80% and a local recurrence rate of 20%. Excellent outcomes have been reported following Moh's surgery, with patients in remission after five years of follow-up.

Based on the literature, the role of chemotherapy in the treatment of EPC remains unclear. Orphan cases with good responses to 5-fluorouracil [14], thiotepa and Cytoxan (cyclophosphamide) [15] have been reported. However, other studies have described cases showing no clinical response to chemotherapy [16]. In one report, four cases of pediatric EPC were treated with a combination of 5-fluorouracil, doxorubicin and cyclophosphamide. No response was observed after one year of therapy [17]. In general, chemotherapy, if considered at all, is reserved for metastatic EPC.

On the other hand, the role of radiation therapy in EPC seems to have changed over the years from radioresistance in earlier reports [15] to good local control in more recent studies [16]. Perhaps the changes in these anecdotal reports reflect the evolution of technological advances in radiation therapy over time. Combinations of photons and electrons were used in several cases. Table 1 summarizes reported cases and outcomes of EPC involving radiation therapy.

**Table 1:** Summary of reported eccrine porocarcinoma cases involving adjuvant radiation therapy.

Location	XRT	Outcome (Remission)	Ref
Hand	70 Gy to tumor and 50 Gy to regional LNs	24 months	[23]
Nose	71.6 Gy to tumor and 50 Gy to supraclavicular LNs	35 months	[22]
Parotid	70 Gy to parotid and 46 Gy to lower cervical LNs	27 months	[22]
Scalp and cervical LNs	76.4 Gy to scalp and 76.5 Gy to posterior cervical LNs	Death at 6 weeks	[22]
Inguinal and para-aortic LNs	50.4 Gy	8 months	[24]
Left vulva	50.4 Gy to left pelvis and 60 Gy to left inguinal LNs	19 months	[3]

LNs: lymph nodes; XRT: radiation therapy.

Until further studies on EPC are conducted, one can extrapolate important lessons from clinical experience with the more common SCC of the head and neck. The parotid gland is a common site for metastasis of cutaneous tumors of the scalp, frontotemporal and periauricular regions. Poor outcomes have been associated with parotid disease, with a two-year survival of only 74%. Survival of patients drops further to 67% when parotid disease is concurrent with neck disease [18]. Patients who require parotidectomy are more likely to have recurrent lesions. Given the importance of parotid involvement, some authorities have advocated modifying the current American Joint Commission on Cancer (AJCC) staging for metastatic cutaneous carcinoma in order to distinguish parotid from neck diseases [19]. Post-operative radiation therapy has emerged as an integral part of the care for aggressive cutaneous carcinoma patients with parotid invasion. A landmark study by Taylor *et al*. demonstrated that patients treated with post-operative radiation therapy had 89% local disease control, compared to 63% for those treated with surgery alone and 46% for those treated with radiation alone [8]. In a more recent study, Weber and colleagues further confirmed the value of a dual approach of surgery and radiation therapy [20].

## Conclusion

More than 45 years after its original description by Pinkus and Mehregan [21], guidelines for staging and treatment EPC are still lacking. The current report describes the presentation and management of an interesting case of cutaneous EPC with metastasis to the parotid gland. Although the short follow-up period is a limitation, to the best of our knowledge there has only been one previously reported case akin to ours [22]. Because of its aggressive potential for metastatic spread, a multidisciplinary approach involving surgery, pathology and radiation therapy should be considered in the management of EPC.

## Abbreviations

AJCC: American Joint Commission on Cancer; CEA: carcinoembryonic antigen; CT: computed tomography; EPC: eccrine porocarcinoma; HIV: human immunodeficiency virus; IMRT: intensity-modulated radiation therapy; PAS: periodic acid-Schiff; PET: positron emission tomography; SCC: squamous cell carcinoma.

## Consent

Written informed consent was obtained from the patient for publication of this case report and any accompanying images. A copy of the written consent is available for review by the Editor-in-Chief of this journal.

## Competing interests

The authors declare that they have no competing interests.

## Authors' contributions

YHZ collected the data regarding our patient, prepared the manuscript, and also researched the related literature. AJZ assisted in interpreting the data from our patient. AKS and EJL supervised YHZ and AJZ and were major contributors in writing the manuscript. MB provided the pathology figures and their legends. All the authors read and approved the final manuscript.
